# Supplementary material for: Second-Line Treatment of Metastatic Renal Cell Carcinoma in the Era of Predictive Biomarkers
Source: Diagnostics (Basel). 2023 Jul 20;13(14):2430. doi: 10.3390/diagnostics13142430 (PMC10378702; doi:10.3390/diagnostics13142430)
Supplement: Supplementary file 1 [file diagnostics-13-02430-s001.zip › diagnostics-2487095-supplementary.pdf]

Table S1: The clinical and biological parameters of patients with mCCRCC

Patient and disease characteristics	Count	Count %
**Patients treated with second line therapy**	**74**	
**All patients Age (years)**	**62.8 (range 43–88)**	
**Gender**		
**Male**	**52**	**70.3%**
**Female**	**22**	**29.7%**
**Surgical treatment**		
**Radical nephrectomy**	**48**	**64.8%**
**Tumour biopsy**	**11**	**14.8%**
**Partial nephrectomy**	**15**	**20.2%**
**The main sites of metastasis**		
**Lungs**	**17**	**23%**
**Distant lymph nodes**	**10**	**13.5%**
**Liver**	**29**	**39.2%**
**Bones**	**21**	**28.4%**
**Fuhrman grade**		
**2**	**35**	**47.3%**
**3**	**33**	**44.5%**
**4**	**6**	**8.1%**
**Karnofsky Performance Status**		
**<80%**	**17**	**23%**
**≥80%**	**57**	**77%**
**Time since diagnosis to treatment <12 months**	**52**	**70.3%**
**≥12 months**	**22**	**29.7%**
**Haemoglobin**		
**< lower limit of normal**	**41**	**55.4%**
**≥ lower limit of normal**	**43**	**44.6%**
**LHD**		
**≥1.5× upper limit of normal**	**12**	**6.2%**
**<1.5× upper limit of normal**	**62**	**83.8%**
**Serum-corrected calcium**		
**≥ upper limit of normal**	**16**	**21.6%**
**< upper limit of normal**	**58**	**78.4%**

**Platelets**		
**≥ upper limit of normal**	**11**	**14.9%**
**< upper limit of normal**	**62**	**83.8%**
**Neutrophils**		
**≥ upper limit of normal**	**24**	**32.4%**
**< upper limit of normal**	**50**	**67.6%**
IMDC score		
**Favourable**	**5**	**6.8%**
**Intermediate**	**38**	**51.4%**
**Poor**	**31**	**41.9%)**
MSKCC score		
**Favourable**	**8**	**10.8%**
**Intermediate**	**49**	**66.2%**
**Poor**	**17**	**23%**
